# Lung contusion drives lung injury by modifying miRNA cargo in alveolar small extracellular vesicles

**DOI:** 10.1007/s00011-025-02051-2

**Published:** 2025-05-24

**Authors:** Keita Nakatsutsumi, Dong Jun Park, Wooil Choi, William Johnston, Katie Pool, Jenny Kezios, Raul Coimbra, Brian P. Eliceiri, Todd W. Costantini

**Affiliations:** 1 Division of Trauma, Surgical Critical Care, Burns and Acute Care Surgery, Department of Surgery, UC San Diego School of Medicine, 200 West Arbor Drive, San Diego, CA 92103-8896 USA; 2 Division of Critical Care and Acute Care Surgery, Department of Surgery, University of Minnesota Medical School, 420 Delaware St. SE, MMC 195, Minneapolis, MN 55424 USA; 3 Comparative Effectiveness and Clinical Outcomes Research Center, Riverside University Health System, Loma Linda University School of Medicine, 26520 Cactus Ave, Moreno Valley, CA 92555 USA; 4 Trauma and Acute Critical Care Center, Institute of Science Tokyo Hospital, 1-5-45 Yushima, Bunkyo-ku, Tokyo, 113-8510 Japan

**Keywords:** Acute lung injury, Bronchoalveolar lavage fluid, Extracellular vesicles, Lung contusion, Micro RNA, Trauma

## Abstract

**Objective:**

To evaluate the micro-RNA (miRNA) cargo of alveolar small extracellular vesicles (sEVs) after lung contusion (LC), which can contribute to the development of trauma-related acute lung injury (ALI).

**Methods:**

A mouse model of LC was conducted with a controlled cortical impact device. Bronchoalveolar lavage fluid (BAL) was collected 24 h post-injury and sEVs were purified using size exclusion chromatography. sEVs characteristics and miRNA cargo were analyzed with vesicle flow cytometry and sequencing. Macrophages were treated with BAL sEVs in vitro to assess their pro-inflammatory effect.

**Results:**

LC increased lung permeability and caused ALI histologically. LC increased the number of sEVs in the BAL and altered their miRNA cargo. BAL sEVs collected after LC increased pro-inflammatory cytokine release from macrophages.

**Conclusion:**

LC increased the mobilization of sEVs to the alveolar space and modified their miRNA cargo that might contribute to the development of ALI by activating the immune response in macrophages.

## Introduction

Lung contusion (LC) occurs in one-third of blunt chest trauma cases and has high mortality [1]. LC disrupts the alveolar septum and pulmonary interstitium, leading to acute lung injury (ALI) [2]. Despite its prevalence and associated risks, treatment options to prevent ALI are limited, highlighting the need to identify biological mediators and molecular pathways involved in ALI development to discover new therapeutic targets.

Small extracellular vesicles (sEVs), also known as exosomes, play a major role in intercellular communication and are associated with the pathogenesis of inflammatory airway diseases [3]. Micro RNA (miRNA) contained within alveolar sEVs causes lung inflammation by increasing the release of inflammatory mediators from pulmonary immune cells [4]. Thus, alveolar sEVs and their miRNA cargo are likely critical in trauma-induced ALI, but the heterogeneity of sEVs following trauma remains insufficiently studied.

The present study aims to evaluate alveolar sEVs heterogeneity and their micro-RNA (miRNA) cargo after LC. We hypothesized that LC alters miRNA cargo in sEVs, which contributes to the development of ALI via increased proinflammatory activity.

## Methods

### Animal models

Male 8–10 week-old C57BL/6J mice were randomized into sham and LC groups. LC mice were anesthetized and subjected to a controlled cortical impact device (Leica Microsystems, Deerfield, IL, USA) to the right chest (velocity: 6.0 m/s, depth: 10 mm) [5]. At 24 h post-injury, lung and bronchoalveolar lavage fluid (BAL) were collected to score ALI histologically [1] and assess lung permeability using the BCA Protein Assay Kit (Cat#A55865, Thermo Fisher, Carlsbad, CA, USA).

### Single vesicle flow cytometry (vFC) analysis

Size exclusion chromatography with a qEV1 column (Cat# ICI-70, IZON Science LTD, Portland, OR, USA) was utilized to isolate sEVs from the mouse BAL supernatant. sEVs concentration and expression of surface proteins were analyzed by vFC using a commercial assay based on a fluorescent lipophilic membrane dye, vFRed (Cellarcus Biosciences Inc., La Jolla, CA, USA) and PE-conjugated antibodies, with a CytoFLEX flow cytometer (Beckman Coulter, Brea, CA, USA) [6]. The following antibodies were used for analysis: CD9, CD63, and CD81 (Cat#130-123-052; 130-123-289; 130-102-632, Miltenyi, San Diego, CA, USA), All flow cytometry data was analyzed using FCS Express (Version 7, De Novo Software, Pasadena, CA, USA).

### miRNA sequencing

RNA, including miRNA, was extracted from alveolar sEVs using miRNeasy (QIAGEN) and prepared for sequencing with Illumina’s TruSeq small RNA protocols. Quality control was performed using an Agilent Bioanalyzer High Sensitivity DNA Chip, and sequencing was conducted on Illumina’s Hiseq 2500 system (Illumina, San Diego, CA, USA). Raw reads were processed to remove unwanted sequences and mapped to miRBase 22.0 to identify miRNAs using the program ACGT101-miR (LC Sciences, Houston, TX, USA). Differential miRNA expression was analyzed using the Student t-test with significance thresholds of 0.01 and 0.05.

### Exposure of alveolar sEVs to macrophages

Alveolar sEVs were added to a mouse macrophage cell line (Raw 264.7) cultured on a 12-well plate. Twenty-four hours after treatment, the cell supernatant was collected, and the concentration of soluble Intercellular Adhesion Molecule 1 (ICAM-1) in the supernatant was evaluated using an Enzyme-linked immunosorbent assay kit (DY450-05, R&D Systems, Minneapolis, MN, USA).

### Statistical analysis

Data were expressed as a mean ± standard deviation. Differences were compared using Student’s t-test. P-values < 0.05 were considered statistically significant. All statistical analyses were performed with Prism 6.0 software (GraphPad Software, La Jolla, CA, USA).

## Results

**Fig. 1:**
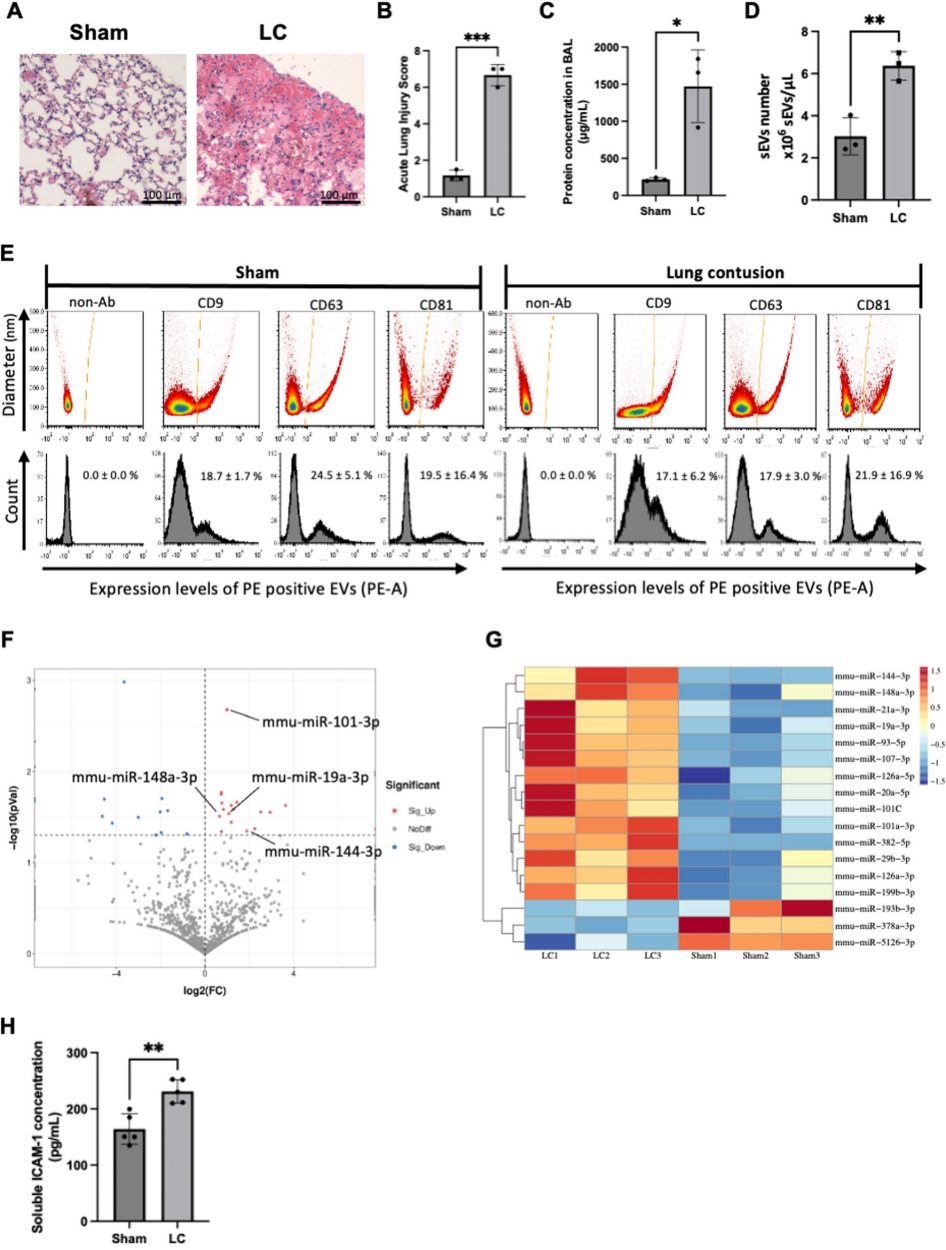
LC increased the histological ALI score and BAL protein concentration (**A**, **B**, **C**). LC increased mobilization of sEVs to the alveolar space (**D**). Alveolar sEVs demonstrated no difference in the expression of canonical EV markers between groups. The representative picture of each group is shown (**E**). miRNA sequencing demonstrated that LC altered the expression of miRNAs (**F**). The heatmap shows miRNAs significantly increased or decreased in alveolar sEVs following LC (**G**). Alveolar sEVs collected after LC increased the concentration of soluble ICAM-1 in the supernatants of macrophages (**H**). Data were presented as mean ± SD (*n* = 3 for each group). BAL, Bronchoalveolar lavage fluid; FC, Fold change; ICAM-1, Intercellular adhesion molecule 1; LC, Lung contusion; miRNA: micro RNA; pVal, *P* value; Sig, Significant; sEVs: Small extracelluar vesicles. *p < 0.05, **p < 0.01, ***p < 0.001

LC increased the histological ALI score (Sham: 1.2 ± 0.3 vs. LC: 6.7 ± 0.6, *p* < 0.01) and lung permeability as demonstrated by increased BAL protein concentration, compared to sham (sham: 216 ± 22 µg/mL vs. LC: 1472 ± 489 µg/mL, *p* = 0.01) (Fig. 1A–C).

LC increased mobilization of sEVs to the alveolar space (sham: 3.0 ± 0.9 × 10^6^ EVs/µL vs. LC: 6.4 ± 0.7 × 10^6^ EVs/µL, *p* < 0.01) (Fig. 1D). Alveolar sEVs demonstrated no difference in the expression of canonical EV markers, such as tetraspanins, CD9, CD63, and CD81, between sham and LC mice (Fig. 1E).

A volcano plot showing the miRNA sequencing results demonstrates that LC altered the expression of several miRNAs within alveolar sEVs (Fig. 1F). The heatmap shows the miRNAs significantly increased or decreased in alveolar sEVs following LC (Fig. 1G).

Alveolar sEVs collected after LC increased the release of soluble ICAM-1 from macrophages in vitro (sham: 164 ± 27 pg/mL vs. LC: 231 ± 21 pg/mL, *p* = 0.01) (Fig. 1H) demonstrating their pro-inflammatory phenotype.

## Discussion

Here, we demonstrated that LC increased the mobilization of sEVs to the alveoli and altered their miRNA cargo, further showing a proinflammatory effect of these LC-derived alveolar sEVs on macrophages in vitro. This alteration of the sEV miRNA cargo after LC can contribute to the development of trauma-induced ALI.

While sEVs in the alveolar space help maintain lung homeostasis in the steady-state, during stress, sEVs are released from pulmonary cells to the alveolar space with altered miRNA cargo, contributing to lung disease [7]. In patients with ALI, sEV levels in the alveolar space have been shown to increase and correlate with disease severity [8]. These sEVs promote macrophage inflammation and disrupt the alveolocapillary barrier by delivering specific miRNAs that drive ALI progression [9, 10].

The miRNAs expressed in alveolar sEVs after LC have been linked to ALI development. miR-144-3p has previously been linked to LPS-induced ALI by activating the JAK/STAT pathway and promoting cytokine production in macrophages [11]. miR-19a-3p has been shown to suppress USP13 expression in sepsis-induced ALI, resulting in increased cytokine production in alveolar macrophages [12]. Additionally, miR-148a-3p has previously been shown to promote M1 macrophage polarization via the PI3K/Akt pathway [13]. miR-101 has also linked to the macrophage immune responses to LPS through the MAPK pathway [14].

Here, we demonstrated that alveolar sEVs collected after LC increased ICAM-1 release from mouse macrophages. Macrophages are known to express ICAM-1 during inflammatory conditions, which is associated with the severity of inflammation [15]. Moreover, increased soluble ICAM-1 in the alveolar space has previously been linked to exaggerated lung inflammation and the development of ALI [16].

In conclusion, our data suggests that LC increased the mobilization of sEVs to the alveolar space and modified their miRNA cargo that might contribute to the development of ALI by activating the immune response in macrophages. Characterizing the miRNA cargo in sEVs recruited to the alveolar space after LC may identify molecular targets to prevent trauma-induced ALI.

## Data Availability

No datasets were generated or analysed during the current study.
